# Counselling in humanitarian settings: a retrospective analysis of 18 individual-focused non-specialised counselling programmes

**DOI:** 10.1186/1752-1505-7-19

**Published:** 2013-09-16

**Authors:** Leslie Shanks, Cono Ariti, M Ruby Siddiqui, Giovanni Pintaldi, Sarah Venis, Kaz de Jong, Marise Denault

**Affiliations:** 1 Médecins Sans Frontières (MSF), Amsterdam, The Netherlands; 2 London School of Hygiene and Tropical Medicine, London, UK; 3 MSF, London, UK

**Keywords:** Mental health, Humanitarian, Médecins Sans Frontières (MSF), Counselling, Conflict, Violence

## Abstract

**Background:**

Médecins Sans Frontières (MSF) provides individual counselling interventions in medical humanitarian programmes in contexts affected by conflict and violence. Although mental health and psychosocial interventions are a common part of the humanitarian response, little is known about how the profile and outcomes for individuals seeking care differs across contexts. We did a retrospective analysis of routine programme data to determine who accessed MSF counselling services and why, and the individual and programmatic risk factors for poor outcomes.

**Methods:**

We analysed data from 18 mental health projects run by MSF in 2009 in eight countries. Outcome measures were client-rating scores (1–10 scale; 1 worst) for complaint severity and functioning and counsellor assessment. The effect of client and programme factors on outcomes was assessed by multiple regression analysis. Logistic regression was used to assess binary outcome variables.

**Results:**

48704 counselling sessions were held with 14963 individuals. Excluding women-focused projects, 66.8% of patients were women. Mean (SD) age was 33.3 (14.1) years. Anxiety-related complaints were the most common (35.0%), followed by family-related problems (15.7%), mood-related problems (14.1%) and physical complaints (13.7%). Only 2.0% presented with a serious mental health condition. 27.2% did not identify a traumatic precipitating event. 24.6% identified domestic discord or violence and 17.5% psychological violence as the precipitating event. 6244 (43.9%) had only one session. For 91% of 7837 who returned, the counsellor reported the problem had decreased or resolved. The mean (SD) complaint rating improved by 4.7 (2.4) points (p < 0.001) and by 4.2 (2.3, p < 0.001) for functional rating. Risk factors for poorer outcomes were few sessions, non-conflict setting (stable or societal violence settings), serious mental health condition, or attending a large, recently opened project.

**Conclusions:**

The majority of clients accessing counselling services present with anxiety related complaints. Attrition rates were high. Good outcomes were recorded among those who attended for more than one visit. Lessons learned included the importance of adaptation of approach in non-conflict contexts such as societal violence or post-conflict contexts. There is a need for further research to evaluate the intervention against a control group.

## Background

Mental health and psychosocial interventions are now a common part of the humanitarian response to war and conflict [1,2]. Despite the proliferation of these interventions, little is known about how the profile and outcomes of individuals seeking care differs across contexts as most publications have described small single-setting mental health programmes [3-5]. Additionally, there is scant information about individual and programmatic risk factors for poor outcomes. There have been recent calls for more research into mental health interventions in humanitarian settings and for humanitarian agencies to introduce rigorous monitoring and assessment of the outcomes of these programmes [6,7].

The majority of research focusing on counselling interventions in humanitarian settings has focused on posttraumatic stress disorder (PTSD). Narrative exposure therapy (NET) is the most studied approach, and where trial designs included a control group, the intervention has been shown to have a positive effect [8-10]. However, a comparison between NET and trauma based counselling in adult refugees did not show a benefit for one intervention over the other, though both did better than the control group [8].

A recent systematic review has reviewed the evidence for psychological interventions in humanitarian settings, and includes a review of the type of intervention provided by Médecins Sans Frontières –Operational Centre Amsterdam (MSF), namely that of focused non-specialised support [6]. Seven randomised controlled trials involving adults were identified for inclusion in the meta-analysis examining the effect of the interventions on PTSD symptoms. The overall result showed a positive impact on symptoms. However, the review did not compare group versus individual counselling, and most of the studies included in the meta-analysis used individual counselling.

MSF mental health programmes consist of individual, group, and community activities integrated into basic health care, initially developed in programmes in Bosnia and Herzegovina [11,12]. As described in these papers, MSF developed the model of intervention based on well known techniques used in resource-rich settings, and used standard instruments validated locally to evaluate outcomes. The initial results were promising and encouraged MSF to continue with the approach. Currently, the psychological component of MSF mental health programmes is mainly delivered through individual sessions, though the actual choice for individual versus group therapy is made by the counsellor at the initial interview in consultation with the client. While group therapy can provide more cost-effective means of reaching the population, in MSF programmes, most clients either request or are referred specifically for individual counselling. One reason for this is that in some of the difficult settings where our programmes are based it is not safe or perceived as safe for individuals to talk openly about their experiences in a group setting.

A standardised registration system has been developed for the MSF counselling intervention based on individual patient-based electronic records, which allows monitoring and evaluation of the programme outcomes. We did a retrospective analysis of individual counselling data collected in MSF mental health programmes in conflict, unstable, post-conflict and societal violence settings during 2009 from eight countries in four continents. We aimed to determine who accessed MSF counselling services and why, and the individual and programmatic risk factors for poor outcomes. We also describe how the results of this analysis were used to adapt the programmes.

## Methods

### Project settings

**Figure 1:**
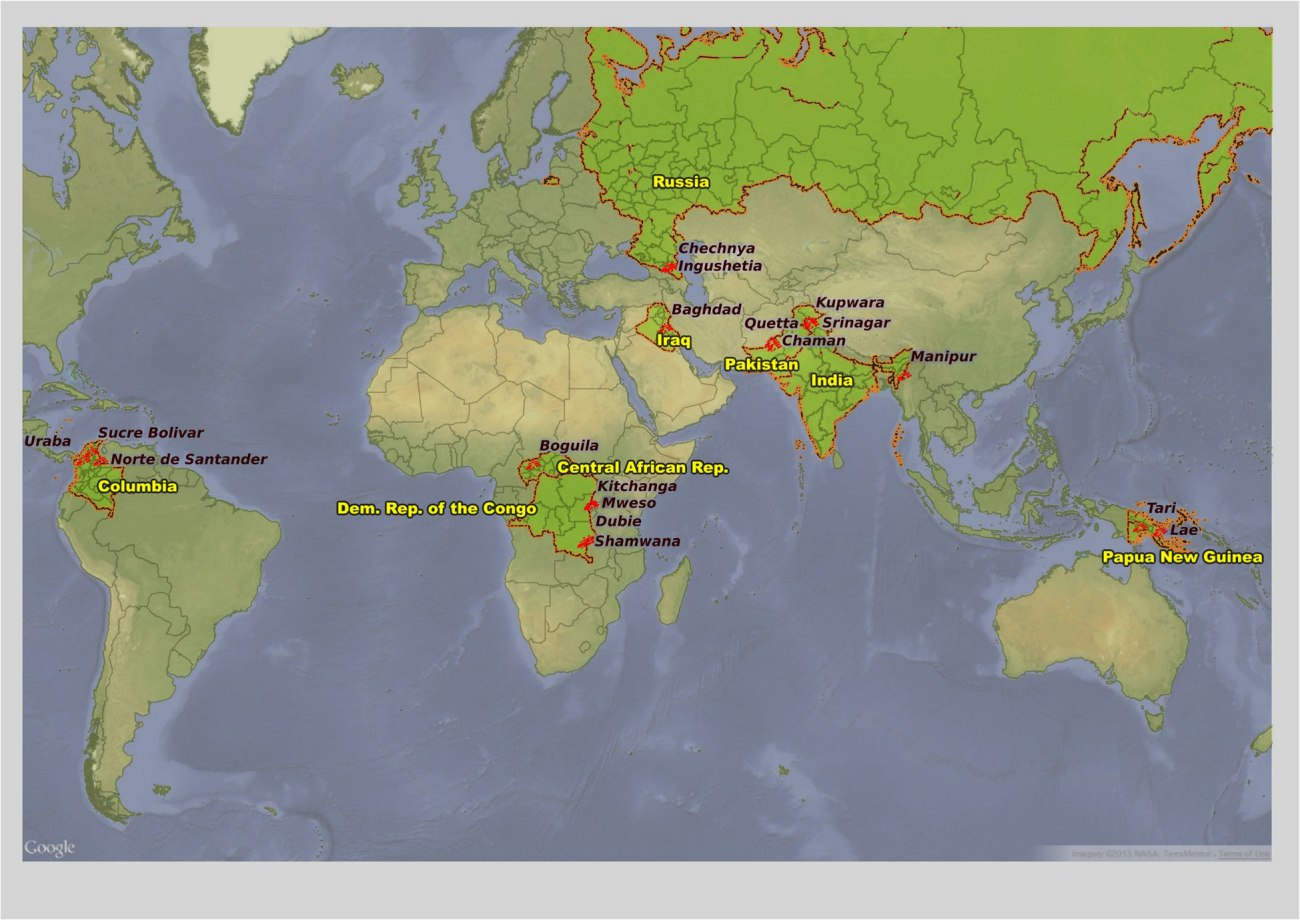
Map of location of MSF mental health programmes.

We included all 18 mental health projects run by the Amsterdam section of MSF in 2009 (Table 1; Figure 1). The setting of each project was classed as conflict, unstable, post-conflict, or societal violence: in 'conflict’ settings there was or had been armed conflict defined as active intra- or interstate conflict in the previous 12 months; 'post-conflict’ settings had a history of armed conflict but no active fighting for at least 12 months; 'unstable’ contexts had political turmoil but the level of violence had not reached an intensity qualifying it as armed conflict; and 'societal violence’ settings had high levels of violence not linked to intra- or interstate conflict or political turmoil.

**Table 1:** Project characteristics

**Project location**	**Context**	**Year project started**	**Number of counsellors**	**Counsellor qualification**
**CAR:** Boguila	Post-conflict	2007	1-3	Lay
**Colombia:**				
Norte de Santander	Conflict	2003	4-6	Academically trained
Sucre Bolivar	Conflict	2005	4-6	Academically trained
Uraba	Conflict	1999	4-6	Academically trained
**DRC:**				
Dubie	Post-conflict	2006	4-6	Lay
Kitchanga	Conflict	> = 2009	≥7	Lay
Mweso	Conflict	> = 2009	1-3	Lay
Shamwana	Post-conflict	2007	≥7	Lay
**India:**				
Kupwara	Unstable	2005	4-6	Academically trained
Srinagar	Unstable	<2000	≥7	Academically trained
Manipur	Unstable	2007	4-6	Lay
**Iraq:** Baghdad	Conflict	2009	4-6	Academically trained
**Pakistan:**				
Chaman	Unstable	2009	1-3	Lay
Quetta	Unstable	2007	1-3	Lay
**Papua New Guinea:**				
Lae	Societal violence	2007	4-6	Lay
Tari	Societal violence	2009	1-3	Lay
**Russia:**				
Chechnya	Unstable	2003	≥7	Lay
Ingushetia	Unstable	2003	≥7	Academically trained

*CAR* Central African Republic, *DRC* Democratic Republic of Congo.

### Intervention

Individuals entered the MSF counselling programme after self-referral or referral by other health professionals. The objectives of MSF’s individual counselling intervention are to reduce suffering and improve functioning rather than to cure people of their problems [13]. The counselling approach is based on principles derived from brief trauma-focused therapy and techniques from cognitive behavioural therapy that are integrated into the cultural context [14,15]. The counsellor seeks to normalise psychosocial reactions, to encourage the expression and containment of emotions and to build resilience and coping skills. The counsellor supports and reinforces the individual’s coping mechanisms through psycho-education, practical advice and helping the client understand the meaning of their experience in the context of their environment. The aim of the counselling intervention is to reduce symptoms and to enhance the client’s functionality through exploration and discovery of new coping mechanisms. The approach is based on the “here and now”, focusing on present difficulties rather than exploring past history unless required by the therapeutic process to achieve the desired outcomes initially agreed by counsellor and client.

Based on the presenting complaint, the counsellor will chose one of six counselling approaches adapted from Van der Veer [16]. For example, a counsellor working with a client experiencing overwhelming feelings will first use psycho-education, explaining that these feelings are normal, and that it is healthy to express them as long as it is done in a controlled way. The counsellor will assist the client in the expression of feelings in the sessions, sometimes using techniques such as drawing or writing exercises. The sessions will also focus on helping the client gain skills in containing their emotions. The complete approach is described in the MSF guideline, *Psychosocial and mental health interventions in areas of mass violence: a community based approach*[17].

Counsellors are locally-recruited and supervised by a professional mental health officer. Where possible, they have an academic background in psychology or social work. If necessary, programmes train lay counsellors who are selected from the local communities. Counsellors receive a standardized 2-week induction course facilitated by experienced mental health officers or psychologists [16]. Standardisation of the counselling intervention is achieved through use of the MSF mental health guidelines [17], annual workshops for mental health officers, oversight from headquarters-based mental health advisors, and weekly on-site clinical supervision from mental health officers. In addition to standardisation of approach and quality control, the clinical supervision aims to provide technical support to the counsellor, assist in overcoming any emotional difficulties hindering the counselling relationship and provide professional education to further develop the skills of the counsellor [18].

Patients are not clinically assessed on entry to the programme, however if counsellors recognize a serious mental health condition they request support from the mental health officer. Treatment of serious mental health conditions (defined according to MSF guidelines as psychosis, delirium, substance abuse, organic brain damage or other) is beyond the scope of the counselling programmes, but in some projects physicians in the primary care services are able to provide psychiatric medications or refer patients to local psychiatrists. Medications are never prescribed by mental health counsellors. The decision to discharge a client is made by the counsellor in agreement with the client based on the counsellor’s judgement of improvement or resolution of the complaint and achievement of the agreed initial goals for the counselling process.

### Data collection

We included all new patients enrolled for individual counselling in MSF routine mental health programmes in 2009. Clinical data were collected at each visit by the counsellor using a standardised client file following MSF guidelines [17]. Data were entered into the electronic database using a client code to protect confidentiality. Anonymised data were sent to MSF headquarters for collation, cleaning and analysis. Data were exported at different times for each project, with the earliest export in February 2010 and the latest in August 2010.

Each individual was identified with a client code. If the client was <5 years, parents received the counselling in order to support their child. Children ≥5 years were counselled directly. Individuals were asked to identify the main complaint that brought them to seek or be referred to counselling. They were asked whether their main complaint could be linked to a specific precipitating event. If the answer was 'no’, it was recorded as 'no traumatic event’. Responses were coded by the counsellor according to standardised categories and sub-categories (Table 2) derived from the Comprehensive Trauma Inventory-104 [19]. How individuals learnt about the counselling service was recorded under 'knowledge of service’. The counselling focus chosen by the counsellor was recorded using six categories adapted from Van der Veer (Table 3) [16]. Exit type was recorded for each closed file. 'Discharged’ was used to refer to discharge from care by the counsellor; 'drop-outs’ referred to individuals who did not return for a scheduled appointment within a specified period of time defined at the project level. Reason for drop-out was included in the exit code.

**Table 2:** Precipitating event

Precipitating event, n = 14808*	**N (%)**
**Conflict and violence**	**4955 (33.5%)**
Psychological violence	**2618 (17.7%)**
Physical violence (intentional)	**196 (1.3%)**
Intentional abuse in detention	**320 (2.2%)**
Witnessing, hearing about abuse, injury or death	**780 (5.3%)**
Displacement, migration and related problems	**401 (2.7%)**
Deprivation or discrimination	**640 (4.3%)**
**Sexual abuse or trauma**	**823 (5.6%)**
**Domestic discord or violence**	**3675 (24.8%)**
**Other precipitating events**	**5355 (36.2%)**
No traumatic event	**4035 (27.2%)**
Separation and isolation	**458 (3.1%)**
Other	**862 (5.8%)**

*155 patients had missing values.

**Table 3:** Counselling focus and associated counselling approach

**Counselling focus**	**Examples of reasons for choosing particular counselling focus**	**Details of the counselling approach**
Practical problems	Lack of information, lack of food and non-food items, tensions or conflicts with other people (such as neighbours and family members)	Help to look at things from a different prospective
		Analysing a recent experience with another person
		Information provision
		Challenging the client
		Clarifying a difficult decision
Lack of skills	Lack of social skills needed to make new friends after separation from family members	Role play to develop social skills
		Provide suggestions to help develop skills
Trauma focused	Physical complaints for which a doctor cannot find causes, or symptoms such as nightmares, anxiety attacks or sudden unexpected outbursts of anger	Choosing target symptoms to focus on
		Assess coping strategies
		Identification and avoidance of triggers
		Psychoeducation to understand origin of symptoms
		Talking about painful past experiences
		Talking about content of dreams
Overwhelming feelings	Overpowering feelings of sadness, anger, etc.	Assist in expression of feelings
		(Drawing/writing)
		Containment of emotions (experiencing and expressing emotions in a controlled way)
Psychiatric	Clients with diagnosed major psychiatric disorder on medication and under care of physician	Counsellor support for taking medication, checking side-effects, education for family
Inner problems	Persistent negative self-view or inner conflict (wanting intimacy yet being afraid to become close due to fear of loss)	Helping clients to recognise and clarify the conflict
		Exploring client’s wishes and the feelings connected to these wishes
		Point out contradictions between what the client is saying, the feelings they have been expressing and their actions

Adapted from: Van der Veer G: *Training counsellors in areas of armed conflict within a community approach*. Utrecht: Pharos Foundation; 2001.

### Outcome measures

Three outcome measures were used. 'Status at last visit’ was scored by the counsellor at the end of each follow-up visit determining whether the presenting problem had resolved, decreased, remained at the same level, or increased in severity. 'Complaint rating difference’ and 'functional rating difference’ were scored by the client: at the first session, the client was asked to score (on a scale of 1 to 10, with 1 being the worst) the severity of their main presenting complaint and the severity by which this complaint reduced their daily functioning. Scoring was repeated at the start of each session. The difference between scores on the last and first visits gave the outcome measures. Outcome analysis included all clients who had attended more than one session and for whom the number of sessions attended had been recorded.

### Data analysis

The descriptive analysis was done using frequency tables and univariate statistics to describe client characteristics. Categorical variables were analysed using Pearson’s *χ*^2^ test and continuous variables with Student’s t-tests.

Independent variables were divided into client and project variables and examined in multiple regression models. Associations between changes in complaint rating, functional rating and status at last visit and independent variables, and the relative predictive importance of independent variables were determined with linear regression. Variables were fitted to a linear regression model and the adjusted R^2^ calculated to measure the amount of variability they explained. P-values from the linear regression F-test were calculated to measure the univariable strength of association. A final model was created combining the strongest predictors from both the project and client variables using multiple regression analysis to evaluate the multi-variable associations between the outcome (difference in complaint rating at the last visit versus the first visit) and the project and client variables. Tests were done for linearity, auto-correlation (Durbin-Watson statistic), homoskedasticity (residual plots), co-linearity (variance inflation factors for the independent variables) and normality of the error distribution (normal probability plot for residuals) and outliers. Robust standard errors were used to correct for heteroskedasticity induced by clustering within country and sites. As similar results were obtained using difference in complaint rating, difference in functional rating or status at last visit, we have presented results only for difference in complaint rating.

For binary outcome variables in both the descriptive analysis and the number of sessions analysis, the associations with independent predictors were assessed using logistic regression with robust standard errors. In particular, four binary outcome variables of the precipitating events were created: conflict and violence (combining categories of physical violence [intentional]; psychological violence; intentional abuse in detention; witnessing, hearing about abuse, injury or death; displacement, migration and related problems; deprivation or discrimination); sexual abuse or trauma; domestic discord or violence; and a category for other types of precipitating events with no direct link to conflict or violence (separation and isolation, no traumatic event and 'other’). Each derived binary outcome used a logistic regression with robust standard errors to measure its association with the context variable. The context category of 'societal violence’ was excluded from this analysis as by definition domestic discord or violence dominated this category and thus distorted comparisons.

The association between the presenting complaint of serious mental health condition and context was analysed by fitting a logistic regression with context setting as the independent variable. The context of societal violence was excluded from the analysis because there was only one individual with a serious mental health condition. Adjustment was done for clustering by project.

Data were analysed using Stata/IC 11.1 for Windows.

### Ethics review

The study received ethical approval from the MSF Ethics Review Board.

## Results

**Figure 2:**
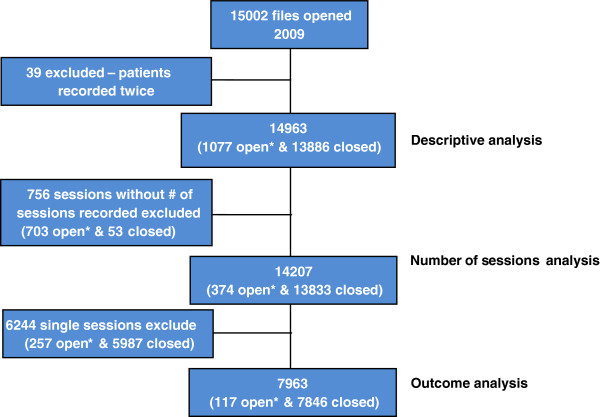
**Decision tree.** *Open files refers to files without date of closure recorded.

In 2009, 15002 files were opened; 39 were duplicates, leaving 14963 individuals in the descriptive analysis (Figure 2). Totals in sub-analyses vary due to missing data. Two projects targeted women clients, Lae (Papua New Guinea) and Chaman (Pakistan). Excluding these, 66.8% (8087 of 12101) of all clients were women; the percentage of women ranged from 56% (93 of 166) to 92.5% (149 of 161) in individual projects. Mean (SD) age was 33.3 (14.1) years (Additional file 1). Although none of the projects specifically targeted children, 1775 clients (11.9%) were younger than 18 years with mean (SD) age of 13.1 (4.2) years. The complete age group distribution included in the descriptive analysis can be found in Table 4.

**Table 4:** Client age group distribution

**Age group (years)**	**Number**	**Frequency**	**Cumulative frequency**
<5	83	0.55	0.55
5-12	574	3.84	4.39
12-16	636	4.25	8.64
16-18	482	3.22	11.86
≥18	13188	88.14	100.00
Total	14963	-	-

### Knowledge of mental health services

Referral source was recorded for 14914 individuals. Clients learned about the counselling services mainly through primary health care clinics (29.1%; n = 4340) and word of mouth (25.4%; n = 3788). Other sources were community activities executed by MSF (17.3%; n = 2580), secondary and tertiary care referrals (16.7%; n = 2491), mass media (5.1%; n = 761), and other (6.5%; n = 969). Note that referrals from health centres include both those run by MSF and those managed by other partners.

### Main presenting complaint

The main presenting complaint as defined by the client was recorded for 14887 individuals. Anxiety-related complaints were the most common reason to seek counselling (35.0%; n = 5207), followed by family-related problems (15.7%; n = 2339), mood-related problems (14.1%; n = 2105) and physical complaints (13.7%; n = 2043). Only 2.0% (n = 304) presented with a serious mental health condition (Table 5).

**Table 5:** Main presenting complaint

Main presenting complaint, n = 14887*	**N (%)**
Anxiety-related	5207 (35.0%)
Family-related	2339 (15.7%)
Mood-related	2105 (14.1%)
Physical complaints	2043 (13.7%)
Behaviour-related	1132 (7.6%)
Loss/mourning	924 (6.2%)
Other serious mental health conditions**	304 (2.0%)
Other	833 (5.6%)

*76 patients had missing values. **Includes psychosis, delirium, substance abuse, organic brain damage.

Most of the 5207 clients who had anxiety-related problems were sub-categorised as having “fear and anxiety, intense psychological distress or hyper vigilance” (38.2%; n = 1989) and/or “worrying” (20.4%; n = 1062). The most common family-related problems were “domestic violence (of client or other family member)” (26.7%; n = 625) and “family discordance/tension” (24.3%; n = 568). The most common mood-related problem was “sadness for long time during the day for several weeks” (39.3%; n = 827). Physical complaints were mainly categorized as “multiple physical complaints or aches” (37.4%; n = 764) and “unclear single complaints or aches” (19.7%; n = 402).

Logistic regression showed no evidence of an association between serious mental health condition as presenting complaint and the context setting (p = 0.384, n = 11510).

### Precipitating event

The most common precipitating event identified by the 14808 individuals for whom this information was recorded was domestic discord or violence (24.6%; n = 3675). 27.2% (n = 4035) did not link any traumatic event to their main presenting complaint. Psychological violence was a precipitating event for 17.5% (n = 2618) (Table 2). The most common sub-categorisation of psychological violence was “being in an area of active conflict, but you were not actively participating and were not injured” (20.4%; 534 of 2618). In the category of domestic discord or violence, only 7.5% (276 of 3675) attributed this event to war or conflict.

In logistic regression analysis there was no evidence of an association between programme context and the grouped precipitating events of conflict and violence (p = 0.848), domestic discord and violence (p = 0.149) or “other” precipitating events (p = 0.552). There was strong evidence of an association with sexual abuse (p < 0.001). Post-hoc analysis showed that the odds of sexual abuse in a conflict setting were 11 times higher (95% CI: 4.30-28.17, p < 0.001) than in post-conflict and unstable settings. There was no difference between the post-conflict and unstable settings for the likelihood of sexual abuse (p = 0.321).

### Severity of complaints at presentation

Table 6 shows the mean severity of complaint and functional ratings at presentation. Regression analysis showed the context was strongly associated with complaint rating at the first visit (p < 0.001). In post-hoc testing, the context of societal violence had lower intensity of complaints on the first visit than the other three contexts (1.19 [95% CI: 0.90 to 1.47, p < 0.001]).

**Table 6:** Distribution of mean (SD) of complaint and functional ratings for each project*

**Project**	**Complaint rating**			**Functional rating**		
	**First visit**	**Last visit**	**Difference**	**First visit**	**Last visit**	**Difference**
**CAR:** Boguila	2.7 (1.9)	7.9 (2.2)	5.2 (2.5)	4.4 (2.7)	8.2 (2.2)	3.9 (2.8)
**Columbia:**						
Norte de Santander	3.5 (1.7)	7.0 (1.6)	3.5 (1.9)	5.0 (2.4)	7.4 (1.9)	2.4 (1.8)
Sucre Bolivar	3.2 (2.4)	7.3 (2.6)	4.0 (2.6)	3.3 (2.5)	7.3 (2.6)	4.0 (2.7)
Uraba	2.7 (1.4)	7.4 (2.3)	4.7 (2.6)	3.4 (1.7)	7.0 (2.3)	3.6 (2.9)
**DRC:**						
Dubie	2.0 (0.9)	6.2 (2.5)	4.2 (2.6)	3.1 (1.7)	6.6 (2.4)	3.5 (2.5)
Kitchanga	2.2 (1.0)	8.8 (1.4)	6.6 (1.7)	3.5 (1.4)	9.1 (1.3)	5.6 (1.7)
Mweso	1.4 (0.7)	5.9 (2.1)	4.5 (2.1)	1.7 (1.0)	6.1 (2.0)	4.4 (2.1)
Shamwana	1.9 (0.6)	7.3 (1.6)	5.4 (1.7)	3.1 (1.1)	7.6 (1.6)	4.6 (1.8)
**India:**						
Kupwara	3.6 (1.2)	6.2 (1.8)	2.7 (1.5)	3.7 (1.2)	6.6 (1.6)	2.8 (1.6)
Srinagar	2.0 (0.8)	6.3 (2.3)	4.3 (2.2)	2.4 (0.9)	6.8 (2.1)	4.4 (2.1)
Manipur	2.6 (1.7)	5.8 (2.4)	3.2 (2.5)	3.2 (2.1)	6.2 (2.4)	3.0 (2.4)
**Iraq:** Baghdad	2.7 (1.1)	7.3 (1.6)	4.6 (2.0)	2.8 (1.0)	7.4 (1.7)	4.6 (1.9)
**Pakistan:**						
Chaman	4.4 (1.5)	7.6 (2.5)	3.2 (2.2)	4.6 (1.4)	7.6 (2.4)	3.0 (2.3)
Quetta	2.7 (1.3)	8.1 (2.3)	5.3 (2.4)	4.3 (1.7)	8.5 (2.0)	4.2 (2.3)
**Papua New Guinea:**						
Lae	3.4 (1.8)	6.0 (2.4)	2.6 (2.6)	4.3 (2.2)	6.5 (2.4)	2.2 (2.6)
Tari	3.2 (1.3)	6.4 (2.3)	3.2 (2.2)	4.0 (1.8)	6.7 (2.1)	2.7 (2.3)
**Russia:**						
Chechnya	2.4 (0.7)	7.9 (1.1)	5.5 (1.2)	2.7 (0.9)	8.2 (1.0)	5.5 (1.2)
Ingushetia	2.2 (1.1)	7.4 (1.3)	5.2 (1.5)	3.1 (1.5)	7.8 (1.4)	4.7 (1.4)
**Overall**	2.6 (1.4)	7.3 (2.1)	4.7 (2.4)	3.4 (1.7)	7.6 (2.1)	4.2 (2.3)

*Scale is 1–10, with 1 the worst/most severe. *CAR* Central African Republic, *DRC* Democratic Republic of Congo.

### Focus of counselling

The most common focus of the counselling intervention in the 14662 individuals for whom this information was recorded was on overwhelming feelings (36.8%; n = 5384). Next most common were trauma-related symptoms (17.5%; n = 2568), lack of skills (16.3%; n = 2382) and practical problems (15.8%; n = 2314). In only 2.2% (n = 305) of cases did the counselling focus on psychiatric support.

### Type of exit

53.6% (7369 of 13736; range across projects 8.3% to 98%) of all clients with type of exit recorded were discharged by the counsellor, 41.4% dropped out, 3.9% re-located and 1.1% of exits were classified as 'other’. Among those who dropped out, 83.6% (4751 of 5683) left without giving a reason or were not able to be traced. Others dropped out because they were feeling better (11.8%), had different expectations or had only required information (4.3%) or were dissatisfied with the service (0.2%). 8.2% of all patients (1227 of 14963) did not have a recorded exit type.

### Number of sessions

The number of sessions was recorded for 14207 clients (Figure 2) who had 48704 sessions. 6244 (43.9%) clients had only one session. The percentage of single session clients in each project ranged from 0.6% to 85.6%. In eight of the 18 projects, >50% of clients attended only one session.

After adjusting for clustering by project, single session clients had an mean complaint rating of 3.17 vs 2.57 for clients with multiple sessions (on average higher by 0.60 [95% CI: 0.21 to 0.99], p = 0.005, n = 13583). Mean functional ratings were 3.87 for single vs 3.39 for multiple session clients (on average higher by 0.48 [95% CI: -0.06 to 1.02], p = 0.079, n = 13580).

A median of two sessions were held per client (IQR 1–5; mean 3.4, SD 3.2; range 1–28 sessions). Mean (SD) time between the first and last session was 68.7 (58.7) days. Lay counsellors had an average of 2.5 more sessions with their clients than did academically-trained counsellors (4.3 vs 1.8, p < 0.001).

Individuals for whom the presenting complaint was domestic discord or violence had significantly fewer sessions than other clients (2.5 vs 3.8 sessions, p < 0.001) as did clients who did not report a traumatic precipitating event (2.5 vs 3.8 sessions, p < 0.001). Clients who reported witnessing or hearing about abuse, injury, or death had a higher number of sessions (5.5 vs 3.3 sessions, p < 0.001).

### Outcome measures

7963 clients had more than one session and were included in the outcome analysis (Figure 2). 305 (3.8%) of those included were <12 years of age and 32 (0.4%) were <5 years.

For 91.0% (7132 of 7837) of clients, the counsellor reported that at last visit the problem had decreased or was completely resolved. 9.0% (705 of 7837) of all clients showed no improvement or worsening complaints; most of these (58.6%) were in Lae (Papua New Guinea). In 1.8% of cases (138 of 7837) the counsellor reported an increase in problem severity.

**Figure 3:**
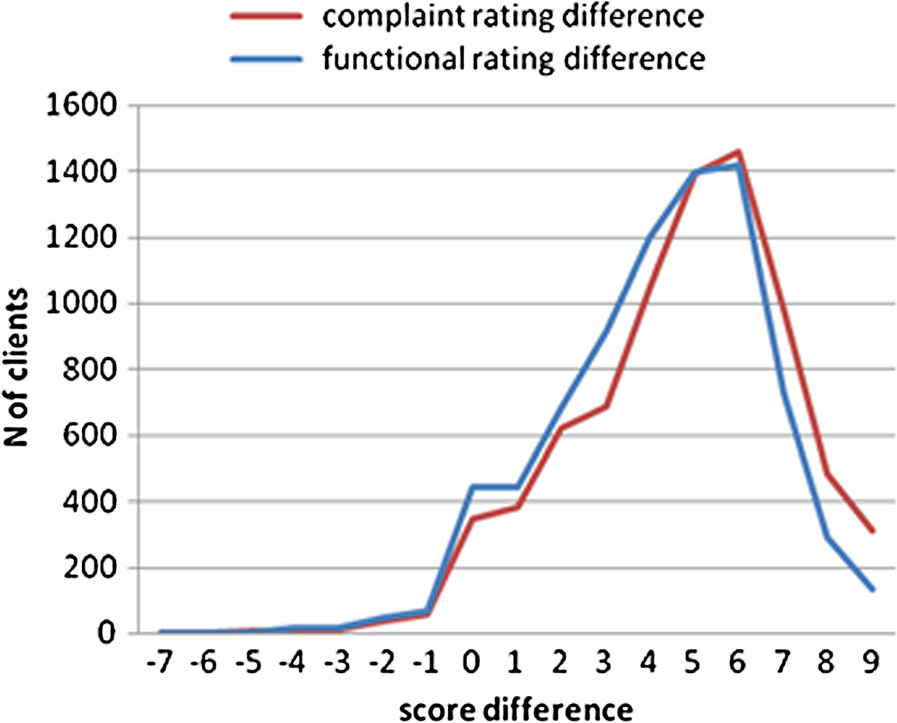
**Distribution of change in rating score between first and last visit.** Rating scores are on scales of 1–10 with 1 the worst/most severe.

Complaint rating at last session across all projects was significantly improved compared to the first session by a mean of 4.7 points (SD 2.4, p < 0.001; Table 6). Similarly functional rating between first and last session improved by 4.2 points (SD 2.3, p < 0.001). Figure 3 shows the distribution of the complaint and functional rating differences.

The model developed in multivariable analysis explained nearly 50% (R^2^ =0.495) of the variability in complaint rating difference (Table 7). The total number of sessions was a strong predictor of outcome with an improvement of 2.07 points (95% CI: 1.51 to 2.63) for roughly each additional 2.7 sessions. The complaint rating at first visit was also a strong predictor (note: the higher the rating at the first visit the smaller the average difference because the rating scale has a fixed maximum of 10).

**Table 7:** Multiple regression of combined model with the difference in complaint rating as dependent variable (n = 7582)

**Variable**	**Coefficient (95%CI)**	**p**
*Log (total sessions)	2.07 (1.51, 2.63)	<0.001
Complaint rating at first visit	-0.62 (-0.72, -0.51)	< 0.001
Age of client (per 10 years)	-0.08 (-0.16, 0.01)	0.061
Context setting		
Societal violence (ref)	0.00	
Conflict	1.30 (1.05, 1.56)	
Post-conflict	-0.08 (-0.58, 0.42)	
Unstable	0.40 (-0.11, 0.90)	< 0.001
Counselling focus		
Practical problems (ref)	0.00	
Trauma-related symptoms	-0.01 (-0.22, 0.21)	
Overwhelming feelings	0.26 (0.04, 0.48)	
Lack of skills	-0.16 (-0.40, 0.08)	
Inner problems	0.01 (-0.8, 0.17)	
Psychiatric support	-0.91 (-1.37, -0.45)	0.007
Serious mental health condition		
Not present (ref)	0.00	
Present	-0.76 (-1.24, -0.29)	0.003
Project size (number of counsellors)		
1-3 (ref)	0.00	
4-6	-1.11 (-1.84, -0.34)	
≥7	-1.06 (-1.49, -0.63)	<0.001
Age of the project		
Commenced prior to 2008 (ref)	0.00	
Commenced in or after 2008	-0.85 (-1.36, -0.35)	0.003

*The total number of sessions showed a logarithmic relationship with the outcome variable and was thus re-coded to its natural logarithm. A client who had had 28 sessions (an outlier) was excluded.

Context was an important predictor of outcome. All else being equal, the outcome was better in conflict and unstable environments than in societal violence and post-conflict settings (p < 0.001). Post-hoc analyses showed that conflict and unstable settings had better outcomes than the post-conflict setting (p = 0.003) and that the conflict setting had better outcomes than the unstable setting (p = 0.002).

Counselling focus was also a predictor of outcome. All else being equal, the outcome was poorest for the psychiatric support focus with a mean worsening of 0.91 points (95% CI: -1.37 to -0.45). In addition, when clients presented with a serious mental health condition the complaint rating difference worsened by 0.76 points (95% CI: -1.23 to -0.29) holding all other variables constant.

Newer projects (started after 2008) showed poorer outcomes with a reduction of 0.85 points (95% CI: -1.36 to -0.34) compared with older projects. Medium and larger projects (4–6 and ≥7 counsellors, respectively) had poorer outcomes than the smallest projects (p < 0.001). Post-hoc analysis showed that the reduction in outcomes was similar in the medium and larger projects (p = 0.875).

## Discussion

Our analysis of almost 15000 clients gives a comprehensive overview of who accessed MSF counselling services in 18 different humanitarian contexts. Our results show a clear gender bias, in that men were underrepresented. Two of the projects exclusively targeted women. However, all projects, irrespective of target group, had a lower percentage of men presenting than women, with a mean of 27.5% male patients. This could be a result of gender-related cultural norms, but could also represent a lack of attention to this group in community education or unrecognised barriers to attracting men [20].

The most common reason for seeking care was 'anxiety-related symptoms’, a significant proportion of which was linked to arousal symptoms such as hypervigilance that are associated with PTSD. Few clients presented with a serious mental health condition, despite associations between psychiatric disorders and violence and war [21,22]. Furthermore, there was no link between more violent project contexts and presenting complaints of a serious mental health condition. Our programmes however did not target major psychiatric disease and did no active case finding.

MSF mental health programmes are designed for those affected by conflict and violence. But our results show that counsellors focused on trauma-related symptoms in fewer than 1 in 5 cases. Only half those enrolled gave a clear history of violence as the precipitating event for their complaint. A large proportion of clients (25%) presented with a precipitating event of domestic discord or violence; few (7%) could directly link this to war or conflict. It may be that underlying disruption to the family caused by the conflict was indirectly linked to the main complaint or that the client was not able to recognise this connection. There was no link between active conflict settings and a precipitating violent event. However the high relative rate of sexual violence found in conflict settings is consistent with the many reports of sexual violence as a weapon of war [23,24].

Outcomes for the clients who returned for a second session were positive, regardless of whether the outcome measure was a client-rated scale or counsellor assessment. Equally important, the percentage of individuals whose scores worsened during treatment was below 2%. This is reassuring given the concern that some forms of psychological intervention after a traumatic event can cause psychological harm [25]. Children were included in the analysis as they are included in our routine programmes and registered and treated using similar principles as in adults. It is unlikely that their inclusion influenced the results; when those under 18 years were excluded from the outcomes model there were no substantial changes.

As others have noted, the number of sessions was strongly linked to successful outcome of treatment [26-28]. We saw a high rate of attrition, though it was highly variable between programmes. In most cases there was no reason given for not returning, but there was an association with increased severity of complaint on presentation, indicating this needs further exploration. In some projects, there were physical, social or even security barriers that prevented clients from returning. People may have been displaced during treatment, or travel may have been insecure. In project sites in Pakistan, women needed to be accompanied by a male family member to leave the home, which may have had an impact on their retention in care. In Colombia, services were often delivered by mobile clinics in very remote, insecure areas, where access was possible only by boat or foot. We are not aware of any other reports of attrition rates in mental health programmes in humanitarian settings. However, in western settings there are reports of high drop-out rates and high rates of patients not returning after the first encounter [26]. Baekeland reported 20-57% of single session visits, compared with our rate of 44% [27]. This suggests that our drop-out rate is not unusual especially given the potential difficulties in our settings with returning for follow-up care.

We were unable to assess outcomes in the 44% of our clients who attended only one session. The impact of a single session of counselling is controversial given the strong association between completed treatment and outcomes [28]. In Colombia almost 95% of individuals reported that a single session was beneficial [29]. In Australia (North Yarra Community) 90% regarded single-session counselling as useful [30]. In Melbourne, Australia, 78-81% were satisfied with single-session counselling as an alternative to being waitlisted for family therapy [31]. However, the MSF model was designed to be a multi-session intervention and was not adapted for single session therapy.

In many resource-limited settings, academically-trained counsellors are not available. Although we were not able to directly compare the quality of care provided by specialised versus non-specialist providers, our findings suggest that lay counsellors can achieve similar outcomes to those academically trained, within the limitations of the outcome measures we used. This is consistent with other positive results with trained lay counsellors [8,32].

Clients with the best outcomes attended more sessions, lived in a conflict setting, were treated in a smaller project more than a year old and did not have a serious mental health condition. Stated differently, risk factors for poor outcomes were attending few sessions, living in a stable setting or one with high levels of societal violence, having a serious mental health condition, and attending a large, recently opened project.

The better outcomes in conflict and unstable settings may be because our mental health programmes are designed for these settings. In contexts where clients had mainly social problems or in post-conflict settings, the reasons for presenting may have been less acute and therefore more difficult to address. Counsellors did not provide psychotherapy. Their aim was to reduce symptoms and improve functionality. Context was also strongly associated with severity of complaint rating at first visit, with societal violence having the strongest association with severity of complaint. Further study is needed to evaluate the best intervention approach in post-conflict and societal violence settings.

Few patients with a serious mental health condition accessed counselling, and those that did derived less benefit than other clients. It is important to note however that our counsellors were not trained to diagnose psychiatric illnesses, nor was the programme designed to target those with severe mental health disorders.

Smaller projects might have had better outcomes because counsellors received more clinical supervision from their mental health officer. The poorer outcomes in newer projects may be due to lack of experience of the counselling staff. MSF invests in training counsellors in a specific approach, and whether lay counsellor or academically-trained, it is likely that the counsellors are more effective with greater experience.

### Limitations

There are several limitations to our analysis which uses routinely collected programmatic data. The data come from a range of programmes, and although a standardised database was used, data quality can vary. Additionally, although intervention approaches were standardised, the individual counsellors or mental health officers could have modified the standard approach. Another potential source of bias is that data were extracted at different times. This is however unlikely to have had a major effect, as the number of sessions analysis included only 2.6% of open files, and only 19 of 7793 open files were included in the outcomes analysis for complaint rating difference. Re-running the outcomes analysis without the open files had a trivial effect on model parameters.

A limitation for the outcomes analysis is that the monitoring tools have not been externally validated. Further, the client rated tools were adapted to the specific cultural context and counsellors were taught to check for understanding, but this might not have been done consistently across and within programmes. Outcomes were consistent between client and counsellor and functional and complaint ratings, suggesting that the results are robust. Nevertheless, the outcome measures are subject to related biases and are interdependent. Finally, as no control group was used, it is possible that the outcomes are due to evolution over time rather than the counselling intervention. This natural improvement over time may also differentially select for acute problems over more chronic issues and may account for the differential improvement in areas of acute conflict as compared to post conflict areas [33]. However this would not account for the poorer outcomes in the context of societal violence where the frequency of acute violent events is also high. Finally, and importantly, we are unable to assess the outcomes in the 44% of clients who did not return for a second visit.

### Lessons learned

The analysis of clients and their outcomes resulted in a number of lessons learned for MSF, many of which may be applicable to other programmes offering individual counselling services in similar settings. The fact that men are significantly under-represented in our programmes, suggest that pro-active targeting of men is needed. This should be supplemented by sensitising medical staff to the importance of identifying men who present in need of mental health counselling. In projects with high numbers of single sessions, especially those due to contextual reasons that are difficult to influence, tailored single-session counselling should be offered. Tailored single sessions would mean less in-depth assessment (of root causes of complaints) and more emphasis on a problem-focused approach where the objective is to provide direct support to the client [34]. This approach has now been adopted by MSF in programmes with high attrition rates after the first session. Results are difficult to measure but feedback from counsellors is thus far positive. In programmes where the attrition rates cannot be attributed to contextual factors, increased attention to adherence is important and should include simple strategies such as reminder phone calls prior to appointments. Finally, perhaps the most important lesson learned is counselling interventions need adaptation to the context. Post conflict settings may demand different intervention models than those in acute conflict or where there is a high incidence of domestic violence. For example in Papua New Guinea where clients present with a history of extreme societal violence, often involving intimate partner violence, MSF has adapted its model of care to include couple’s counselling and anger management training.

## Conclusions

Within the limits of our outcome measures, our programme analysis demonstrates that good outcomes can be achieved for those who return for a second visit in our programmes over a variety of humanitarian contexts where MSF intervenes. Client-related risk factors for poor outcome were a serious mental health condition and fewer sessions. Programme or context-related risk factors were post-conflict or stable settings with societal violence, larger programmes, and new programmes.

We identified a number of areas which merit further research. Strategies to improve the uptake of men into mental health programmes and to reduce the high rate of attrition need to be designed and evaluated. In addition, our positive programmatic outcomes suggest that a clinical trial of the effectiveness of the intervention against a control group is merited.

Lessons learned included that in programmes with high attrition rates, it is important to offer an adapted single session intervention and improve adherence strategies. The most important lesson learned was that one size does not fit all, and adaptation of approach is needed particularly in non-conflict settings such as societal violence or post-conflict contexts.

## Competing interests

The authors declare that they have no competing interests.

## Authors’ contributions

LS: Designed the original study, contributed to the analysis, wrote the first draft of the paper and approved the final version. CA: Performed the final analysis, contributed to writing the paper and approved the final version. GP: Contributed to data analysis and commented on earlier versions of the paper, approved final version of paper. SV: Contributed to the analysis and writing the paper and approved the final version. KJ: Designed the original registration system and approved final version of the paper. RS: Contributed to the analysis and writing the paper and approved the final version. MD: Contributed to data collection and data analysis, and commented on earlier versions of the paper, approved the final version of the paper. All authors read and approved the final manuscript.

## Supplementary Material

Additional file 1 Client profiles by project. Click here for file
