# Supplementary material for: Counselling in humanitarian settings: a retrospective analysis of 18 individual-focused non-specialised counselling programmes
Source: Confl Health. 2013 Sep 16;7:19. doi: 10.1186/1752-1505-7-19 (PMC3849884; doi:10.1186/1752-1505-7-19)
Supplement: Additional file 1 — Client profiles by project. [file 1752-1505-7-19-S1.doc]

Table 4: Client profiles by project

| **Project** | **N** | **Men, n (%)** | **Women, n (%)** | **Mean age (SD) years** |
| --- | --- | --- | --- | --- |
| **CAR:** Boguila | 793 | 261 (32.9%) | 532 (67.1%) | 33.2 (11.0) |
| **Colombia:** |  |  |  |  |
| Norte de Santander | 636 | 202 (31.8%) | 434 (68.2%) | 25.2 (15.0) |
| Sucre Bolivar | 1243 | 368 (29.6%) | 875 (70.4%) | 27.8 (15.6) |
| Uraba | 378 | 104 (27.5%) | 274 (72.5%) | 26.9 (15.0) |
| **DRC:** |  |  |  |  |
| Dubie | 615 | 237 (38.5%) | 378 (61.5%) | 37.5 (13.3) |
| Kitchanga | 926 | 170 (18.4%) | 756 (81.6%) | 40.7 (14.3) |
| Mweso | 101 | 42 (41.6%) | 59 (58.4%) | 35.3 (13.7) |
| Shamwana | 289 | 118 (40.8%) | 171 (59.2%) | 44.0 (13.9) |
| **India:** |  |  |  |  |
| Kupwara | 992 | 381 (38.4%) | 611 (61.6%) | 33.6 (14.6) |
| Srinagar | 1920 | 814 (42.4%) | 1106 (57.6%) | 29.6 (13.1) |
| Manipur | 268 | 70 (26.1%) | 198 (73.9%) | 34.6 (12.6) |
| **Iraq:** Baghdad | 166 | 73 (44.0%) | 93 (56.0%) | 31.9 (13.5) |
| **Pakistan:** |  |  |  |  |
| Chaman | 358 | 1 (0.3%) | 357 (99.7%) | 38.8 (11.5) |
| Quetta | 1178 | 361 (30.6%) | 817 (69.4%) | 38.2 (14.9) |
| **Papua New Guinea:** |  |  |  |  |
| Lae | 2495 | 102 (4.1%) | 2393 (95.9%) | 28.9 (10.0) |
| Tari | 161^1^ | 11 (6.8%) | 149 (92.5%) | 26.1 (13.2) |
| **Russia:** |  |  |  |  |
| Chechnya | 1418^1^ | 524 (37.0%) | 887 (62.6%) | 40.0 (12.5) |
| Ingushetia | 1026^1^ | 278 (27.1%) | 747 (72.8%) | 37.7 (12.9) |
| **Total** | **14963** | **4117 (27.5%)** | **10837 (72.4%)** | **33.3 (14.1)** |

CAR=Central African Republic. DRC=Democratic Republic of Congo. The following projects have patients with missing values for gender: Tari (1), Chechnya (7) and Ingushetia (1).
